# Robust inflammatory and fibrotic signaling following volumetric muscle loss: a barrier to muscle regeneration

**DOI:** 10.1038/s41419-018-0455-7

**Published:** 2018-03-14

**Authors:** Jacqueline Larouche, Sarah M. Greising, Benjamin T. Corona, Carlos A. Aguilar

**Affiliations:** 1 Dept. of Biomedical Engineering, University of Michigan, Ann Arbor, MI USA; 2 School of Kinesiology, University of Minnesota, Minneapolis, MN USA; 3 Wake Forest University School of Medicine, Winston-Salem, NC USA; 4 Present Address: Australian Regenerative Medicine Institute, Monash University, Melbourne, Australia

Skeletal muscle has a remarkable regenerative capacity, which is conferred by a pool of resident stem cells, known as satellite cells. After damage, satellite cells proliferate, differentiate, and fuse to form new or repair existing multinucleated myofibers. However, after surgical or traumatic loss of a critical mass of muscle, also known as volumetric muscle loss^1^ (VML), this endogenous regenerative competence is overwhelmed. Rather VML has been shown to induce robust scar deposition, fibrotic supplantation, loss of function, and serious morbidity^2^. These outcomes have been postulated to result from the ablation of resident regenerative progenitors in addition to connective tissue and basement membrane, which provide structural, biochemical, and mechanical cues to guide regeneration^3^. Regenerative therapies that aim to restore these elements, such as autologous tissue or stem cell transfer^3^ from an uninjured site, or implantation of an instructive scaffold^4^ that recruits and guides reparative cells, have yielded incomplete recovery of muscle volume, strength, and function.

The development of successful regenerative therapies for VML has been hindered by an incomplete understanding of the molecular phenomena driving and mediating injury repair. In this issue of *Cell Death and Discovery*, Aguilar et al.^5^ addresses this issue by characterizing the pathophysiologic response to VML using a multi-scale approach, and contrasting those results to surgical implantation of a regenerative therapy (minced muscle grafts-MMGs). The investigators tracked the molecular phenomenology after VML over 56 days using muscle function testing, histology, and gene expression profiling using high-throughput sequencing (RNA-sequencing). Consistent with previous reports, histological analysis showed progressive fibrosis, macrophage infiltration, and minimal muscle fiber regeneration throughout the period observed^6, 7^. Using RNA-seq on the injured tissues and several types of bioinformatics analyses, the investigators found a series of enriched gene sets associated with chemotaxis and inflammation that were followed by pathways associated with excessive extracellular matrix (ECM) deposition and remodeling. These results were in contrast to many muscle regenerative studies^8^, where inflammatory pathways subsided after several days^9^. Instead, VML injury appears to stimulate complement, Wnt and TGF-β signaling in a sustained fashion, which in turn activates fibrosis development. These pathways, coupled with inefficient debris clearance, have been shown^10^ to influence the actions of multipotent mesenchymal progenitors, called fibro-adipogenic progenitors (FAPs), triggering their proliferation and differentiation into fibroblasts or adipocytes^11^ and their production of excess matrix.

**Fig. 1:**
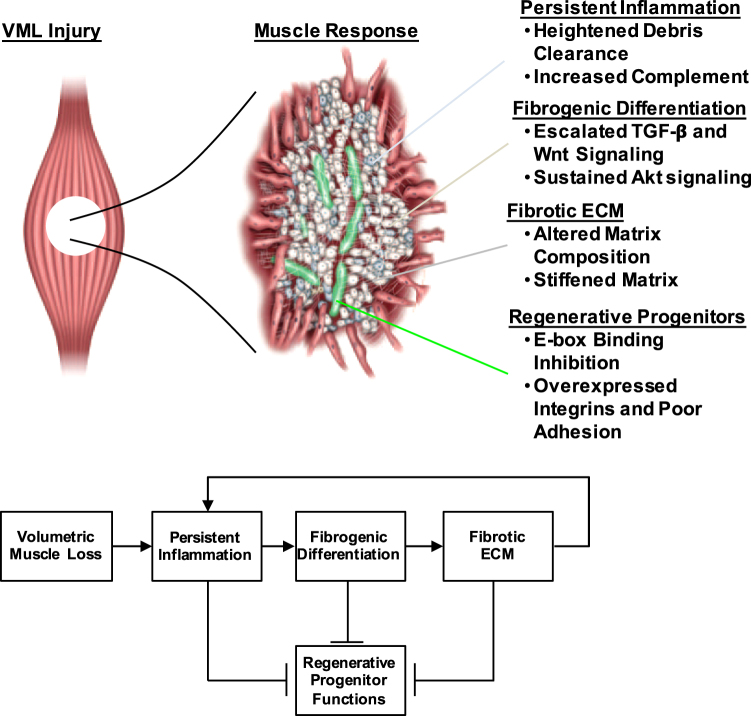
Volumetric muscle loss (VML) is proposed to induce a barrier to muscle regeneration through a feedback loop driven from inflammation and fibrosis. The activation of this feedback loop prevents the reparative activities of myogenic progenitors on multiple levels and prevents appropriate healing

Uniquely, when VML was treated with MMGs, the transcriptional landscape of the tissue did not vary considerably and the deleterious pathways described above were marginally affected. The authors described multiple programs that could be contributing to this effect, including a sustained inflammatory response, dysregulated and stiff ECM (which would confer alterations in integrin signaling), as well as increases in expression of transcription factors (Smad2/3, Snai1, Id2, Id3, Bmp1) that block differentiation-promoting myogenic transcription factors (MyoD, MyoG, Mef2). Aguilar et al. then stipulate that VML injury drives muscle into a myogenesis-inhibitive feedback loop^12^, where perturbations, such as those delivered from MMGs, do not impact the fibrotic outcome (Fig. 1). One factor contributing to formation of this inhibitive feedback loop (and limited effectiveness of therapies) is the composition and duration of the immune cell presence at the injury site. Therapeutic modalities that incorporate immunomodulatory elements^13^ may promote a more favorable environment for subsequent regenerative therapies, by removing debris and emitting soluble factors that recruit and support regenerative progenitors. For example, Corona et al.^14^ administered Tacrolimus, a calcineurin phosphatase inhibitor that reduces macrophage and dendritic cell activity and inhibits interleukin-2 (IL-2) mediated activation of T lymphocytes, in combination with MMGs. Administration of systemic Tacrolimus with MMGs reduced the functional deficit by about one third compared to MMGs alone. A deleterious effect of persistent inflammation that contributes to and perpetuates the inhibitive regenerative feedback loop is the production of a fibrotic environment^11^. Recently, it has been shown in a model of Duchenne muscular dystrophy (DMD), where chronic damage is observed, that inflammatory cells regulate the activity of FAPs and induce their differentiation into collagen-producing fibroblasts and adipocytes^12^. The persistent inflammatory and fibrotic environment observed after VML mimics this pathology and alters the behavior of muscle progenitor cells, immune cells, and pathologic fibroblasts to adopt a degenerative phenotype. A mediator of this phenomenology is TGF-β1, which is a popular target for treatment of fibrosis, and is observed to be dysregulated after VML injury. Together, this suggests that the development of a combinatorial approach incorporating immunomodulatory/anti-fibrotic elements followed by the delivery of reparative muscle components, including satellite cells, may be effective for treatment of VML. Such an approach would also uniquely resist fibrosis (since healthy myogenic progenitors emit exosomes that restrain collagen biosynthesis)^15^, aid in the preservation of remaining tissue, and be able to accommodate future regenerative demands.

To date, several groups have used different kinds of regenerative medicine strategies to treat VML, such as cell transplants, ECM scaffolds, physical therapy, and combinations^6^ of these. However, all of these approaches failed to fully restore function and may be due to the fact that the underlying molecular landscape is not impacted by these therapeutic methods. Numerous gaps still exist for VML, such as the myogenicity of remaining cells in the defective area, the degree to which transplanted cells engraft, and the evolution of the intramuscular nerves and vasculature in the defect site. Each of these features can impact functional recovery and necessitate further investigation. Overall, the data-centric approach used to understand the transcriptional networks induced from VML and development of therapies targeting these networks described by Aguilar et al. could be useful in a range of applications. While the transcriptome profiling approach used whole tissue samples, which only provides an average transcriptional signature and does not reflect individual components (or antagonism between compartments), many of the gene targets discovered by Aguilar et al. have also been discovered to be dysregulated in DMD and aging. Sorting individual populations of cells (and potentially profiling single cells from each population) should overcome this challenge and provide additional insights into effectors of tissue mechanics, inflammation and restraint, integrin evolution, and myogenic potential providing a plethora of translational opportunities.
